# Prosthetic Soft Tissue Management in Esthetic Implant Restorations, Part I: Presurgical Planning, Implant Placement, and Restoration Timing. A Narrative Review

**DOI:** 10.1002/cre2.900

**Published:** 2024-11-08

**Authors:** Faezeh Atri, Kimia Nokar

**Affiliations:** ^1^ Department of Prosthodontics, School of Dentistry, Craniomaxillofacial Research Center Tehran University of Medical Sciences Tehran Iran; ^2^ Department of Prosthodontics, School of Dentistry Tehran University of Medical Sciences Tehran Iran

**Keywords:** Dental Implants, Dental Prosthesis, Implant‐Supported, Esthetics, Dental, Immediate Dental Implant Loading

## Abstract

**Objectives:**

This two‐part review article delineates various techniques to enhance esthetic outcomes in anterior implant treatments. Part I concentrates on presurgical measures, case selection, implant placement, and restoration timing. Part II discusses post‐surgical steps, the temporary restoration phase, the emergence profile contour, abutment types, and impression techniques.

**Material and Methods:**

A comprehensive search was conducted using Medline/PubMed, Embase, Scopus, and the Cochrane Library. The primary keywords included were “Dental Implants,” “Dental Prosthesis, Implant‐Supported,” “Esthetics, Dental,” “Dental Impression Techniques,” and “Tissue Management.”

**Results:**

Initially, 1472 studies were identified, from which 187 were selected based on publication year and title relevance. After removing duplicates, 84 abstracts were reviewed in full text, culminating in 59 studies being thoroughly analyzed.

**Conclusions:**

Optimal esthetics in implant restorations are attainable through meticulous treatment planning, precise surgical execution, and systematic restorative steps, ensuring predictable outcomes. Factors such as a thick gingival biotype, an intact facial bone wall, and atraumatic extraction significantly contribute to superior esthetic results. Immediate implant placement combined with immediate provisionalization provides the most predictable outcomes by supporting and maintaining soft tissue architecture. Conversely, delayed implant placement and provisionalization often require extensive manipulation of collapsed soft tissues to achieve desired esthetics.

## 1 Introduction

Replacing teeth in esthetic zones remains a significant challenge within the clinical setting. In 1982, Dr. Branemark introduced benchmarks for successful dental implants, which included minimal bone loss, optimal gingival health, functionality, and patient satisfaction (Brånemark, Zarb, and Albrektsson 1985). By 1989, Smith and Zarb (1989) had established criteria for evaluating implant success, notably emphasizing the importance of an adequate esthetic appearance for the first time. Subsequently, in 1996, Garber (1996) highlighted that the condition of the peri‐implant soft tissue is a crucial determinant of the esthetic outcome, and Besler suggested that a successful implant‐supported restoration must replicate the appearance of natural teeth (Belser, Buser, and Higginbottom 2004).

Today, therapeutic success in the anterior region is primarily gauged by function, patient satisfaction, and esthetics. To objectively measure esthetics, indices such as the Pink Esthetic Score (PES) and White Esthetic Score (WES) have been introduced (Fürhauser et al. 2005). The white esthetic is achieved through effective collaboration between dentists and technicians. In contrast, the pink esthetic assesses the soft tissue surrounding the implant, considering various factors such as the mesial and distal papilla, soft tissue level, alveolar process deficiency, and the color and texture of the soft tissue (Fürhauser et al. 2005). Creating an appropriate emergence profile and soft tissue contour is predominantly the dentist's responsibility, with the technician needing to adhere to these guidelines during restoration fabrication.

To achieve desirable esthetic outcomes, clinicians must consider multiple factors, including the gingival phenotype (Lee et al. 2020; Meng, Chien, and Chien 2021; Oh et al. 2006), maintenance of the papilla (Tarnow, Magner, and Fletcher 1992), the thickness of the buccal bone wall (Monje et al. 2023), implant insertion techniques (Kan et al. 2018), and the timing of implant and restoration procedures (Kinaia et al. 2017; Pitman et al. 2022). This review article aims to discuss the significance of each factor and provide insights that will help clinicians predict the outcomes of treatments.

## 2 Materials and Methods

A comprehensive search of electronic databases, including Medline/PubMed, Embase, Scopus, and Cochrane, was conducted from January 2008 to May 2023. The search utilized a combination of the following MESH terms and keywords: (“Dental Implants” OR “Dental Prosthesis, Implant‐Supported”), (“Immediate implant” OR “fresh socket” OR “Delayed implant”), “Immediate Dental Implant Loading,” and (“Esthetics, Dental” OR “Tissue management”). We included randomized controlled trial studies focusing on implant placement and restoration in the anterior region. Studies involving complex or novel surgical procedures that addressed the rehabilitation of completely edentulous patients, bone augmentation or grafting, or utilizing the socket shield technique were excluded. Priority was given to systematic reviews, randomized controlled trials, cohort studies, and clinical guidelines. Only studies published in English were considered.

Patients provided informed consent for the treatment and the use of their photographs in publications.

## 3 Results

Initially, 1472 studies were identified; 187 were selected based on the publication year and title relevance. After the removal of duplicate citations, 84 abstracts of full‐text articles were reviewed, culminating in 59 studies being thoroughly examined and included in the analysis.

### 3.1 Presurgical Phase

#### 3.1.1 Soft Tissue Considerations

The esthetic outcome of the treatment heavily relies on the soft tissue's stability. Studies on immediate implants indicate that most soft tissue loss occurs within the first 3 months post‐extraction, stabilizing around 6 months (Tian et al. 2019). These findings were based on a 1‐year follow‐up with a limited sample size.

Several factors, including gingival phenotype, the amount of keratinized tissue, and the flap technique employed, can influence peri‐implant soft tissue stability (Lee et al. 2020; Meng, Chien, and Chien 2021; Oh et al. 2006). A lack of adequate keratinized mucosa often leads to soft tissue inflammation and mucosal recession (Ramanauskaite, Schwarz, and Sader 2022), while a thin gingival biotype may cause buccal soft tissue dehiscence (Sanz‐Martín et al. 2022). Thus, it is recommended to maintain a keratinized gingiva of at least 2 mm width on the buccal aspect and to employ a flapless technique when feasible to optimize esthetic results (Lee et al. 2020; Meng, Chien, and Chien 2021; Oh et al. 2006).

In scenarios involving a thin gingival biotype (Kan et al. 2018) or when an increase in soft tissue thickness is required, concurrently performing a connective tissue graft with either immediate or delayed implantation is advised (Jung et al. 2022; Thoma et al. 2021). One particular study (Fujita et al. 2021) utilizing Cone Beam Computed Tomography (CBCT) assessed the esthetic outcomes of immediate implantation in the esthetic zone with and without guided bone regeneration combined with a connective tissue graft; the findings suggested that the latter approach significantly enhanced soft tissue profiles, potentially compensating for underlying bone loss. However, the limitations of this study were the short follow‐up period and a small patient cohort. Additionally, grafting the gap between the socket and the implant has proven beneficial for maintaining soft tissue integrity, particularly in cases of immediate implant placement (Kan et al. 2018; Seyssens, Eeckhout, and Cosyn 2022).

#### 3.1.2 Interproximal Papilla Maintenance

Papilla formation is influenced by several factors, including the distance between the crestal bone and the proximal contact (Tarnow, Magner, and Fletcher 1992), the nature of adjacent structures (Gholami et al. 2022; Tarnow et al. 2003), the height of the interproximal bone, and crestal bone levels (Tarnow et al. 2003), as well as the morphology of the interproximal space (Agra Souza et al. 2019).

**Figure 1:**
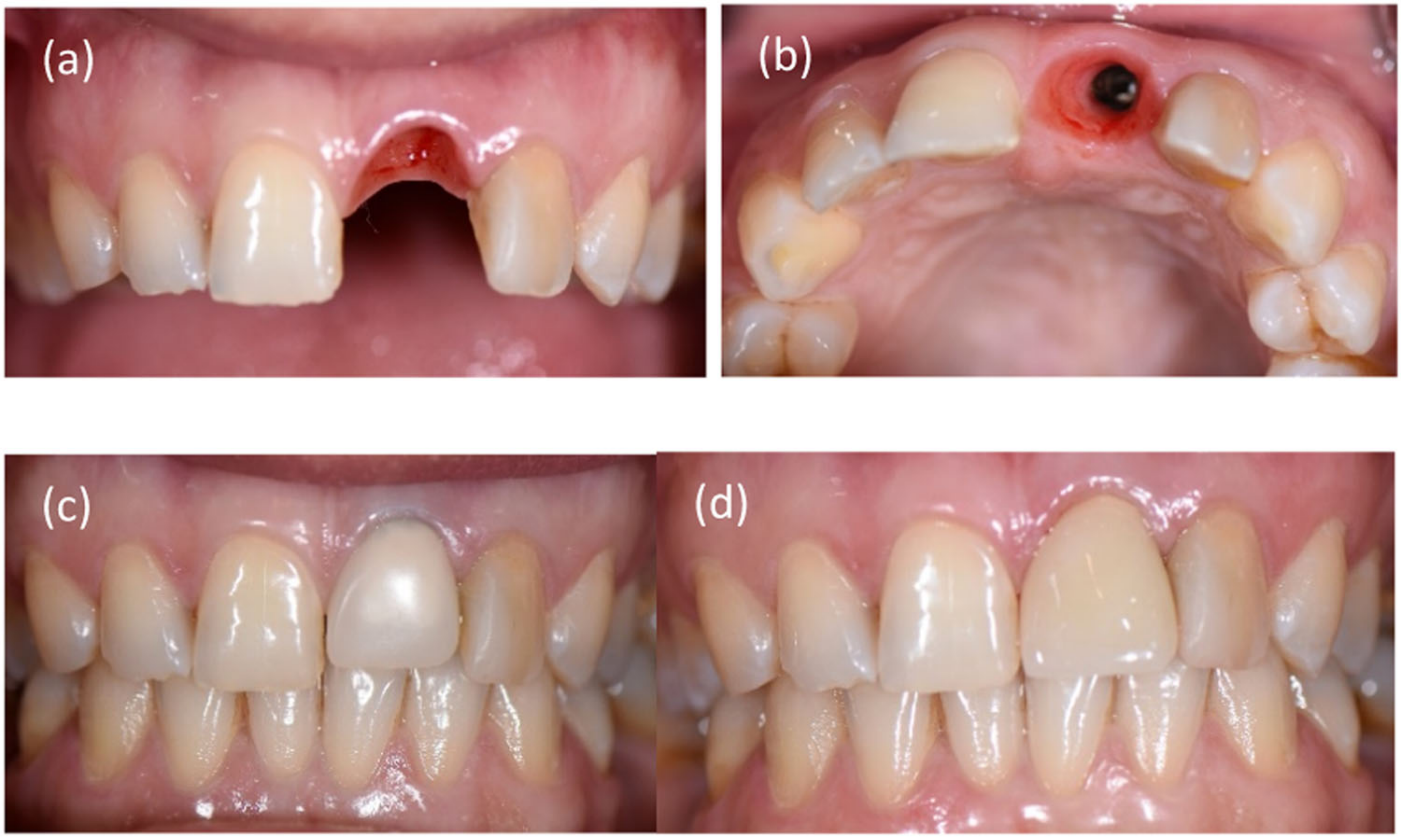
Soft tissue molding and papilla preservation; (a) frontal view of the papilla, (b) occlusal view of formed gingiva, (c) provisional restoration, and (d) final restoration.

Tarnow posited that papilla presence hinges on the gap between the proximal contact point and the bone crest. Specifically, if this distance measures 5 mm or less, papilla presence is assured in 98% of cases. Conversely, a distance of 6–7 mm reduces papilla completeness to 56% and 27%, respectively (Tarnow, Magner, and Fletcher 1992). Adjacent structures also play a critical role. A retrospective clinical study (Gholami et al. 2022) showed that papilla height was maximized between an implant and a natural tooth, assuming integration of the periodontal ligament, compared to configurations involving a pontic or another implant; however, the differences were not marked. Tarnow et al. (2003) observed that the average papillary height between two implants was 3.4 mm. Consequently, replacing two anterior teeth with a single implant is advisable to achieve a higher papilla level, approximately 5.5 mm, between an implant and a pontic (Salama et al. 1998) (Figure 1).

Agra et al. (2019) proposed that the morphology of the interproximal space is a primary determinant in the presence or absence of the interproximal papilla. They deduced that patients with thick gingival biotypes and short, broad papillae tend to retain their papillae more than those with thin gingival biotypes and long interproximal papillae. These findings are insightful; however, the study's cross‐sectional nature and the evaluation of only 144 implants call for further longitudinal studies and comprehensive systematic reviews for validation.

#### 3.1.3 Buccal Bone Wall Thickness

Implant placement in healed ridges typically leads to long‐term resorption and changes in the premaxilla; generally, post‐tooth extraction, bone resorption is inevitable, even with concurrent implant placement (Monje et al. 2023; Raes et al. 2018). This resorption is particularly pronounced in thin gingival biotypes and predominantly affects the buccal bone plate in the premaxilla (Buser et al. 2013).

A systematic review (Monje et al. 2023) assessing the impact of buccal bone wall thickness on soft and hard tissue changes concluded that sites with a thin buccal bone wall (< 1.5 mm) are more susceptible to significant changes, potentially compromising the buccal bone's integrity and causing biological and esthetic concerns. This review was deemed methodologically robust by the Assessing the Methodological Quality of Systematic Reviews (AMSTAR) checklist (Shea et al. 2007), affirming the reliability of the results.

Conversely, some studies suggest that the importance of buccal bone width might be exaggerated (Raes et al. 2018; Sanz‐Martín et al. 2022). An 8‐year cohort study found that a preserved buccal bone alone is sufficient for achieving desirable esthetic outcomes (Raes et al. 2018). The study proposed that a 2 mm buccal bone wall thickness could mitigate bone loss (Merheb, Quirynen, and Teughels 2014), though such thickness is often not attainable in practice. The study further indicated that varying widths of an intact bony wall can still support satisfactory esthetic results, provided the surrounding soft tissue remains healthy. It adheres properly to the implant, thus preventing pocket formation and maintaining stable soft tissue levels, ultimately preserving the esthetics of the treatment.

Further research shows that following tooth extraction, patients with a thin bone wall phenotype often experience an increase in the thickness of the buccal soft tissue, whereas in cases with a thick bone wall, the soft tissue remains unchanged (Chappuis, Araújo, and Buser 2017; Chappuis et al. 2015; Raes et al. 2018).

Various strategies have been recommended to reduce changes in the buccal bone plate and prevent its resorption, including immediate implant placement (Santhanakrishnan et al. 2021), connective tissue grafts (Buser et al. 2013), simultaneous bone augmentation at the time of implantation (Monje et al. 2023) even when the implant is fully embedded in bone (Raes et al. 2018), ridge preservation (Buser et al. 2013), and the use of narrow‐diameter implants and hybrid design (HD) implants in cases of thin ridges (Monje et al. 2023). These approaches should be employed as deemed appropriate by the clinician.

### 3.2 Surgical Phase

#### 3.2.1 Position and Technique of Implant Placement

To properly place an implant, it is crucial first to determine the optimal location, which involves considering the intended position of the final restoration and the quality of the underlying bone. The technique for implant insertion can either be performed freehand or by utilizing a guided approach, which can be either conventional or digital.

In 2018, Kan et al. (2018) provided guidelines for implant placement as follows:

- The implant should be centered under the planned restoration and maintained at a minimum distance of 2 mm from adjacent teeth.
- For optimal stability, the implant should be positioned along the palatal wall of the extraction socket and slightly lingual relative to the final restoration. This buccolingual positioning ensures a space of at least 1.5 mm between the implant and the buccal bone, thus preserving the integrity of the labial bone.
- The implant neck should be inserted approximately 3 mm apical to the future restoration's free gingival margin.

They further recommended that for achieving stability with immediate implant placements, selecting cases with no infection, an intact bony socket with at least 1 mm of buccal bone width, and sufficient bone apical and palatal to the implant are required (Kan et al. 2018).

Midfacial recession is a prevalent issue that can compromise esthetic outcomes. Research indicates that buccal placement of implants significantly increases the risk of mucosal recession, more so than other factors such as thin gingival biotype (Evans and Chen 2008; Sanz‐Martín et al. 2022, 2020). Therefore, opting for a lingual shoulder or cingulum position parallel to the palatal wall is a judicious choice.

Additionally, employing a flapless or papilla‐preserving approach, practicing minimally invasive or atraumatic tooth extraction, maintaining a 2 mm gap between the buccal lamella and adjacent natural teeth, and ensuring a 2 mm distance from the bottom of the socket to the implant tip (for stability) can enhance the esthetic results of immediate implantation (Yan et al. 2016). This meta‐analysis is deemed high quality according to the AMSTAR checklist tool (Shea et al. 2007), which supports the reliability of these findings.

Recent advancements suggest using cutting‐edge virtual implant planning tools that incorporate CBCT data to enhance the predictability and precision of implant placements in the esthetic zone (Meng, Chien, and Chien 2021; Pozzi et al. 2021). However, the choice of technique—whether freehand, using a guided stent, or planning digitally—should depend on the practitioner's expertise and the case's complexity.

An alternative that could potentially improve esthetic outcomes in the anterior upper jaw is using zirconia implants instead of traditional titanium ones (Sivaraman et al. 2018). Nonetheless, caution should be exercised with zirconia implants as, although they have shown similar short‐term clinical results to titanium implants, there are limited data on their long‐term clinical performance (Afrashtehfar and Del Fabbro 2020).

#### 3.2.2 Timing of Implant Placement and Restoration

Regarding the timing, immediate implant placement occurs within 24 h post‐extraction, while delayed implant placement happens within 3–6 months post‐extraction (Francisco et al. 2021). Immediate restoration is performed within 1 week of implant placement, and delayed restoration occurs after 2 months (Gallucci et al. 2018).

Numerous reviews and clinical studies suggest that the esthetic outcomes are not significantly influenced by the timing of implant placement and restoration (Cheng et al. 2020; Francisco et al. 2021; Parvini et al. 2022; Pommer et al. 2021; Yan et al. 2016). Understanding the tissue dynamics influenced by various implant and restoration timing protocols is crucial for informed treatment decisions. Discussion on timing is organized around two clinical scenarios:

1. Immediate implant placement, with or without immediate provisionalization
2. Delayed implant placement, with or without immediate provisionalization

##### 3.2.2.1 Immediate Implant Placement and Esthetic Outcomes

Immediate implant placement in the esthetic zone can preserve gingival contours and papillary architecture, leading to superior esthetic results and minimized buccal bone alterations compared to delayed implant placement (Bhuvaneshwari et al. 2020; Felice et al. 2016; Kan et al. 2018; Santhanakrishnan et al. 2021; Slagter et al. 2021). However, this approach may incur higher risks of implant failure and other complications (Esposito et al. 2015; Felice et al. 2015; 2016; Kan et al. 2018). Ideal candidates are those with thick gingival biotypes.

Post‐immediate implantation, the restoration can be immediate or delayed. The esthetic outcomes tend to be favorable if immediate restoration is feasible. Research consistently shows comparably satisfactory esthetic results for immediate implantation, irrespective of the restoration timing (Chan et al. 2019; Francisco et al. 2021; Hassani, Hassani, and Bitaraf 2021; Slagter et al. 2021). Nevertheless, two recent systematic reviews with high AMSTAR scores (Shea et al. 2007) observed lesser midfacial recession and greater papillary heights with immediate restorations than delayed ones in immediate implants (Kinaia et al. 2017; Pitman et al. 2022). Thus, immediate provisionalization is a viable option when appropriate.

###### Immediate Implant Placement With Immediate Loading

This scenario delivers the most predictable esthetic outcomes. The primary benefits of immediate loading include the support of soft tissues during the healing period, minimized tissue loss (De Rouck et al. 2009; Pitman et al. 2022; Sutariya et al. 2022), and enhanced esthetic results (den Hartog et al. 2016; Kan et al. 2018). This technique also shortens the treatment duration, reduces surgical interventions, and ensures esthetics from surgery day to the final restoration (den Hartog et al. 2016; Pitman et al. 2022).

However, some studies note that immediate loading combined with immediate implantation poses a higher risk of implant failure than immediate loading with delayed implantation, although the esthetic results after 1 year tend to be similar between both approaches (Esposito et al. 2015; Felice et al. 2015; Raes et al. 2018).

Accurate patient selection is paramount; preferred candidates have no history of smoking, no parafunctional habits, no pre‐existing infections, and adequate soft and hard tissue conditions, including at least 1 mm of facial bone thickness and a thick gingival biotype (Kan et al. 2018).

**Figure 2:**
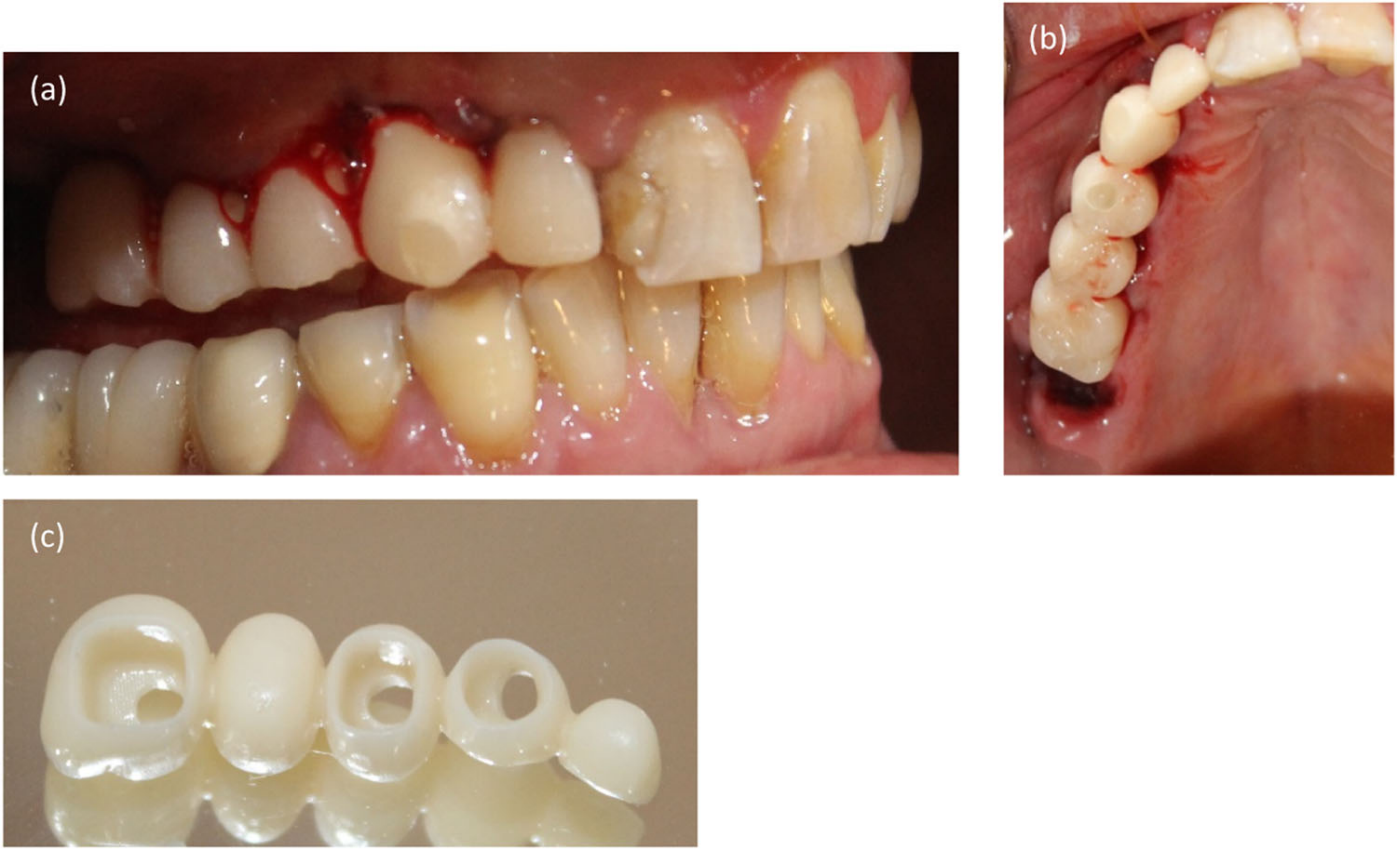
Immediate implant placement and immediate provisional restoration. (a) Buccal view, (b) occlusal view, and (c) cement‐screw provisional restoration.

After implant placement, the clinician must assess the primary stability to decide whether to proceed with immediate restoration (De Rouck et al. 2009). If conditions permit, a provisional restoration should be crafted to sculpt the gingiva and emergence profile akin to the adjacent natural teeth (Figure 2).

###### Immediate Implant Placement With Delayed Loading

Immediate loading is not feasible in certain instances due to inadequate hard tissue for the primary stability of the implant, a substantial gap between the implant fixture and bone socket necessitating extensive grafting, or patients exhibiting parafunctional habits (Gupta et al. 2019; Ilser and Ashley Brooke 2015). Under these circumstances, the implant should be shielded with a healing abutment or a gingival former, coupled with concurrent hard and soft tissue augmentation at the time of implantation (De Rouck et al. 2009; Sutariya et al. 2022). Furthermore, a randomized controlled trial involving 40 patients indicated that in scenarios with insufficient stability at the time of immediate placement, a flared healing abutment yielded results comparable to those of immediate provisionalization (Chan et al. 2019). However, these findings are not universally applicable due to the limited sample size and a follow‐up period of only 1 year.

##### 3.2.2.2 Delayed Implant Placement and Esthetic Outcomes

In situations where delayed implant placement is employed, bone and gingiva tend to resorb, resulting in the loss of the papilla, which complicates achieving satisfactory esthetic results. Delayed implant placement is recommended for young patients who have lost teeth due to trauma, pregnant women, and cases involving large periapical lesions or ankylosed teeth located apically (Buser et al. 2017). An infection at the intended implant site or inadequate biological conditions during extraction time also necessitate a delayed approach (Buser et al. 2017; Gupta et al. 2019).

Two randomized controlled trials comparing immediate non‐occlusal loading with delayed loading in the esthetic zone revealed no significant differences between the two protocols (den Hartog et al. 2011; den Hartog et al. 2016). These findings align with a strong systematic review (using the AMSTAR checklist; Shea et al. 2007), which evaluated 18 trials (Francisco et al. 2021). According to Hartog et al. (2016), since the presurgical soft tissue has already collapsed by the time of delayed implantation, immediate loading does not provide the benefits it might have in cases of immediate implantation.

In delayed implantation, the provisional phase preceding definitive restoration is particularly advantageous, especially in the esthetic zone. It helps guide tissue healing, shape the gingiva, and develop the emergence profile (den Hartog et al. 2016; Furze et al. 2019).

###### Delayed Implant Placement With Immediate Loading

**Figure 3:**
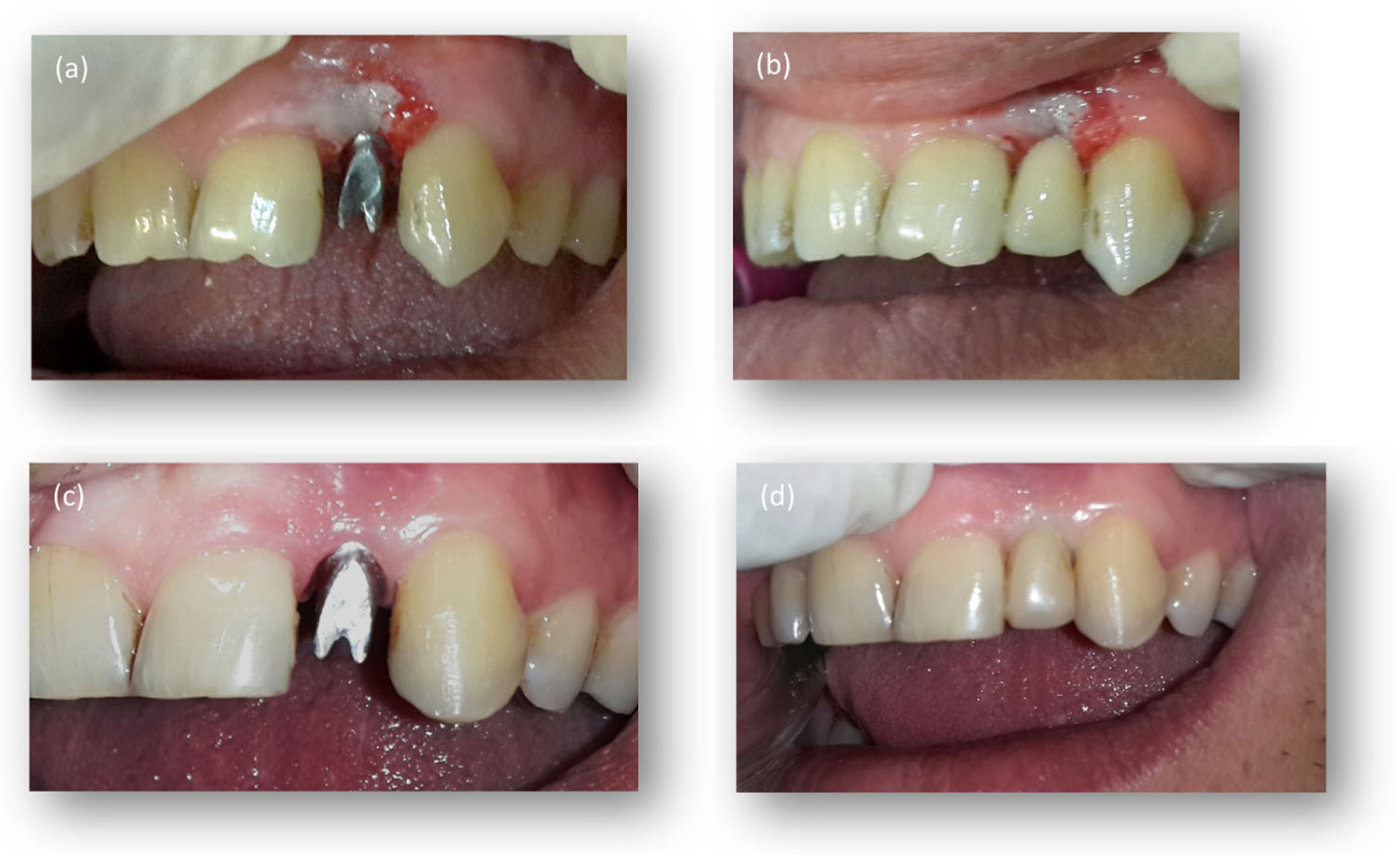
(a) Delayed implant placement. (b) Immediate provisional restoration. (c) Molded emergence profile. (d) Definitive natural‐looking restoration.

Immediate restoration can be undertaken if the implant achieves sufficient primary stability upon insertion. The contour of the provisional restoration should mirror the final restoration, or it may be narrower at implant placement. Subsequently, following osseointegration, the final shape can be refined stepwise (Figure 3).

###### Delayed Implant Placement With Delayed Loading

This scenario typically arises in cases characterized by severe ridge resorption, insufficient soft tissue, significant infection proximal to the implant site, or the necessity for multidisciplinary treatments such as orthodontics. It is also observed in cases involving inappropriate nonanatomic healing abutments and two‐stage implants (Buser et al. 2017; Gupta et al. 2019). The challenge of restoring esthetics predictably is most significant in the context of delayed implant placement combined with delayed loading due to prior extraction. This leads to the resorption of soft and hard tissues and the absence of a provisional restoration to guide tissue healing. Effectively shaping the gingival scallop requires multiple provisional restorations and considerable time.

## 4 Conclusions

Achieving ideal esthetics in implant restorations is contingent upon meticulous treatment planning, precise surgical execution, and methodical steps throughout the restorative process to ensure predictable outcomes. The strategy for treatment hinges on the patient's presurgical conditions.

For immediate implant placement, the optimal candidates possess a thick gingival biotype, an intact facial bone wall with a minimum thickness of 1 mm, and teeth extracted atraumatic. The methodologies for implant placement and the timing for restoration are delineated across four clinical scenarios:

1. **Immediate Implant Placement with Immediate Provisionalization**
  This scenario provides the most predictable esthetic results as immediate restoration supports and stabilizes the soft tissue architecture.
2. **Immediate Implant Placement with Delayed Provisionalization**
  The implant is placed post‐extraction immediately, but the provisionalization is delayed, which may require minimal adjustments to preserve the tissue contour and architecture.
3. **Delayed Implant Placement with Immediate Provisionalization**
  In this approach, the implant placement is postponed, typically to allow for infection resolution or bone graft maturation. However, once the implant is placed, provisionalization is executed immediately to support the soft tissues and maintain esthetic contours.
4. **Delayed Implant Placement with Delayed Provisionalization**
  This represents the most challenging scenario for achieving esthetics, as it involves extensive manipulation of collapsed soft tissues, which must be reconstructed. This approach is often reserved for cases with significant anatomical challenges.

In conclusion, the choice of implant placement and provisionalization timing critically influences the esthetic outcomes, with immediate implant placement and immediate provisionalization offering the most favorable scenario for maintaining soft tissue architecture and achieving optimal esthetics.

## Author Contributions

*Conceptualization:* Faezeh Atri. *Methodology*: Faezeh Atri and Kimia Nokar. *Search and Selection*: Kimia Nokar. *Writing–original draft preparation*: Faezeh Atri and Kimia Nokar. *Writing–review and editing*: Faezeh Atri and Kimia Nokar. *Critical review*: Faezeh Atri. All authors read and approved the final manuscript.

## Conflicts of Interest

The authors declare no conflicts of interest.

## Data Availability

The two‐part review article aimed to explore published studies. Data sharing does not apply to this article as no new data were created or analyzed in this study.
